# Abnormal Early Gamma Responses to Emotional Faces Differentiate Unipolar from Bipolar Disorder Patients

**DOI:** 10.1155/2014/906104

**Published:** 2014-03-13

**Authors:** T. Y. Liu, Y. S. Chen, T. P. Su, J. C. Hsieh, L. F. Chen

**Affiliations:** ^1^Institute of Brain Science, School of Medicine, National Yang-Ming University, Taipei 112, Taiwan; ^2^Integrated Brain Research Unit, Division of Clinical Research, Department of Medical Research, Taipei Veterans General Hospital, Taipei 112, Taiwan; ^3^Department of Computer Science, National Chiao Tung University, Hsinchu 300, Taiwan; ^4^Department of Psychiatry, Taipei Veterans General Hospital, Taipei 112, Taiwan; ^5^Division of Psychiatry, School of Medicine, National Yang-Ming University, Taipei 112, Taiwan

## Abstract

This study investigates the cortical abnormalities of early emotion perception in patients with major depressive disorder (MDD) and bipolar disorder (BD) using gamma oscillations. Twenty-three MDD patients, twenty-five BD patients, and twenty-four normal controls were enrolled and their event-related magnetoencephalographic responses were recorded during implicit emotional tasks. Our results demonstrated abnormal gamma activity within 100 ms in the emotion-related regions (amygdala, orbitofrontal (OFC) cortex, anterior insula (AI), and superior temporal pole) in the MDD patients, suggesting that these patients may have dysfunctions or negativity biases in perceptual binding of emotional features at very early stage. Decreased left superior medial frontal cortex (smFC) responses to happy faces in the MDD patients were correlated with their serious level of depression symptoms, indicating that decreased smFC activity perhaps underlies irregular positive emotion processing in depressed patients. In the BD patients, we showed abnormal activation in visual regions (inferior/middle occipital and middle temporal cortices) which responded to emotional faces within 100 ms, supporting that the BD patients may hyperactively respond to emotional features in perceptual binding. The discriminant function of gamma activation in the left smFC, right medial OFC, right AI/inferior OFC, and the right precentral cortex accurately classified 89.6% of patients as unipolar/bipolar disorders.

## 1. Introduction 

Mood disorders, including major depressive disorder (MDD) and bipolar disorder (BD), are among the most debilitating diseases worldwide and with a lifetime prevalence estimated about 20.8% [1]. Guidelines from the Diagnostic and Statistical Manual of Mental Disorders, fourth edition (DSM-IV) [2], characterize the manic/hypomanic episodes as an index for distinguishing bipolar from unipolar disorders, but misdiagnosis of bipolar as unipolar could occur particularly when the BD patients come to the hospital in depressive episodes [3, 4]. Identification of disorder biomarkers disclosed from neuroimage researches could improve diagnostic accuracy and clinical treatment outcomes of bipolar disorder [4]. Precious neuroimaging studies have proposed that dysfunction of facial expression perception is one of core impairments in the MDD and BD patients [5–8]. Hence quantitative measurements of neural responses to emotional stimulation may facilitate accurate diagnosis and better treatment outcomes of the MDD and BD patients.

Depressed patients have an attentional bias toward negative stimuli (easier to attract patients' attention) [9], which are more inclined to interpret neutral faces as sad [10, 11]. Stimulation with high arousal levels would activate the negative motivational withdrawal system more strongly than the positive approach system [12], which is called the negativity bias [13]. It implicates that withdrawal from negative stimuli is more critical to survival than approach of pleasant or neutral stimuli [13, 14]. To achieve this goal, humans “early” allocate attentional resources to negatively valenced stimuli in an efficient way, as proposed in the automatic vigilance model of emotion [15], which leads to delayed attentional disengagement. Hence we speculate that the depression symptoms in mood disorders may associate with their increased negativity bias, which results in dysfunction of early rapid processing of resources allocation.

Implicit emotional paradigms, by which participants attend to judge nonemotional perceptual features (e.g., gender) rather than emotional cues, have been considered an effective way to explore neural substrates of facial emotional processing [16, 17]. Perceptual processing of emotion-laden stimuli engages several critical brain regions, including the amygdala, prefrontal cortex, orbitofrontal cortex (OFC), anterior cingulate cortex (ACC), and anterior insula (AI) [18]. Previous studies showed that the abnormal activity of these regions in patients with affective disorder may be related to their specific symptoms, including anhedonia (easy to be unhappy and sad) and emotional instability [5]. However, there is limited understanding of dysfunction of neurobiological basis underlying “early” emotion perception. The present study aimed at elucidating whether the MDD and BD patients have impaired or biased perception of emotional facial features at very early stage of emotional processing.

The current study measured gamma activity by magnetoencephalography (MEG) and adopted implicit emotional paradigms to investigate early facial emotion perception in BD and MDD patients, compared with healthy controls and to distinguish these two affective disorders by discriminant analyses. Gamma-band responses have been implicated to be a mechanism of perceptual binding and strongly synchronized about 100 ms after sensory processing, reflecting integration processing of perceived features at very early stage [8, 19, 20]. Müller et al. [21] suggests that spatial distribution of gamma oscillations, including limbic, temporal, and frontal cortices may be linked to neural substrates of binding emotional information. Gamma oscillations can provide a potential index to explore regional brain abnormalities of early emotional processing in affective disorders. Previous studies have showed that subcortical and cortical regions activate within 100 ms by high temporal resolution technique, such as event-related potentials (ERPs) and magnetoencephalography (MEG) [22, 23]. Our previous study also demonstrated alterations of gamma activity (50–150 ms) during early emotion processing in the MDD and BD [7]. We in this study further tested that alterations of cortical gamma responses to early perception of emotional facial expression were distinct between the MDD and BD patients, which can be a potential index to differentiate patients with unipolar from bipolar disorders.

## 2. Materials and Methods

### 2.1. Subjects

Seventy-two participants were recruited from the Department of Psychiatry at Taipei Veterans General Hospital in this study, including twenty-three MDD patients (mean age 35.96 ± 9.58, nine males), twenty-five BD patients (mean age 36.80 ± 11.38, ten males), and twenty-four age- and gender-matched healthy controls (NC, mean age 36.62 ± 11.36, nine males). The three groups did not show significant differences in age (a one-way ANOVA, *P* = 0.961) and gender (2 × 3 contingency table analysis, *P* = 0.916). All subjects were right-handed as assessed by the Edinburgh Handedness Inventory. The diagnosis of MDD and BD was confirmed by a structured interview based on the Diagnostic and Statistical Manual for Mental Disorders (DSM-IV) criteria (American Psychiatric Association, 1994). Before MEG data acquisition, psychiatric and mood symptoms of all patients were assessed with the Young Mania Rating Scale (YMRS) and the 17-item Hamilton Rating Scale for Depression (HAMD). For details, see Table 1. The NC subjects underwent the Mini International Neuropsychiatric Interview before participation in the study to confirm the absence of past or current psychiatric symptoms. Each participant signed informed consent forms approved by the Institutional Review Board at Taipei Veterans General Hospital.

### 2.2. Stimuli and Experimental Design

Face images with duration of 1.5 sec (72 trials for each emotion and four emotions in total, including neutral, sad, happy, and angry faces) were displayed randomly. Visual stimuli were exhibited at the center of a back-projected translucent screen, which was located 100 cm in front of the subject, and subtended 14° (width) by 17° (height) of visual angle. Subjects were instructed to perform a gender discrimination task by lifting their left/right index finger for male/female face images, respectively, while a response cue was displayed. All subjects practiced the test before their MEG signals were recorded.

### 2.3. MEG and MRI Recordings

Event-related MEG data were recorded by a whole-head 306-channel neuromagnetometer (Vectorview 306, Elekta Neuromag, Finland) with a sampling rate of 1000 Hz and a 0.03~330 Hz bandpass filter. Trials containing deflections exceeding 9000 fT/cm or contaminated by eye movements were excluded for the source analysis. The signal space projection method [24] was applied to remove urban and device interference in the recorded MEG data. The T1-weighted MRI (magnetic resonance images) of each individual was acquired by a 1.5 T GE Signa Excite scanner using an 8-channel phased-array head coil with 3D fast spoiled gradient recalled echo (3D FSPGR, TR = 8.67 ms, TE = 1.86 ms, inversion time = 400 ms, matrix size = 256 × 256 × 124, and voxel size = 1.02 × 1.02 × 1.5 mm^3^). To facilitate precise coregistration of the MEG data and structural MRI, three anatomical landmarks (nasion and left and right preauriculars) were localized with Isotrak 3D digitizer (Polhemus Navigation Sciences, Colchester, Vermont, USA).

### 2.4. MEG Source Analysis

For each emotion, the noise-free MEG data were filtered at a frequency band of 35 to 55 Hz (gamma). These gamma-band signals were then analyzed through a beamforming method [25] to estimate cortical activity index of emotional evoked responses, which was denoted as the gamma-band activation index (GBAI). The GBAI map with an isotropic voxel size of 4 mm in the whole-brain was obtained by estimating the ratio between estimated signals of the active state (a 30-ms window) and those of the control state (from 300 ms to 200 ms before stimulus onset) for each voxel. This study focused on investigating the difference of brain responses between patients with MDD and BD in emotion processing during the first 100 milliseconds after stimulus onset (from 30 to 120 ms with 5 ms shift).

For group analysis, the individual T1-weighted MRIs were first transformed into the Montreal Neurological Institute (MNI) space (a standard stereotactic space) with an isotropic spatial resolution of 2 mm by the BIRT software [26]. The obtained deformation field was then applied to transform the individual GBAI maps obtained above into the MNI space for further group analysis. A one-way ANOVA (*F*(2, 69) = 7.65, uncorrected *P* < 0.001, cluster size = 100) and two two-sample* t*-tests (*t*(47) = 3.87 for BD versus NC group,* t*(45) = 3.88 for MDD versus NC group, uncorrected *P* < 0.000167) were conducted at each time point in a voxel-wise manner by using the statistical parametric mapping software (SPM2, http://www.fil.ion.ucl.ac.uk/spm/). The intersection areas between the survived voxels obtained from the ANOVA and* t*-test analyses were extracted and the mean of GBAI within each area with cluster size ≥30 was calculated for the following correlation and discriminant analyses.

### 2.5. Correlation and Discriminant Analyses

Pearson correlation coefficients were used to assess the relationship between the abnormal regional GBAIs and symptomatic/demographic data in patient groups. The correlation was determined to be significant at least 10 ms (three continuous maps, e.g., 30, 35, and 40 ms).

Abnormal regional GBAIs of the MDD and BD patients obtained from the above-mentioned source analysis procedure were extracted as possible features for differentiating these two patient groups. A two-stage discriminant analyses were performed to identify distinguishable feature variables (abnormal regional GBAIs) and their weightings by evaluating their contributions in distinguishing patient groups (MDD versus BD). In the first stage, a stepwise linear discriminant procedure was used to select the predictors of the model, which can be best able to distinguish between these two patient groups. At each step, one variable was considered at a time and Wilks' Lambda values of the variables in the model were used to determine this variable to be a predictor or not. The threshold of Wilks' Lambda was set at 0.25 for setting retention in the model, which was based on previous Monte Carlo simulation studies [27]. This procedure iteratively repeated for each variable until there was no further improvement in discriminability of the model.

The second stage was a canonical discriminant analysis which was used to determine the weightings of those variables selected from the first step. The resolved standardized weight of each variable (regional GBAI) reflected its relative discriminating efficiency. The accuracy of the derived discriminant function from the two-stage discriminant analyses was assessed by leave-one-out cross validation for the whole patient cohort. Furthermore, a two-sample* t*-test was used to test whether the mean values of the discriminant function for the patient groups were different. Finally, a Fisher Exact Test was performed to evaluate the statistical significance of the classification accuracy [28].

## 3. Results 

**Figure 1:**
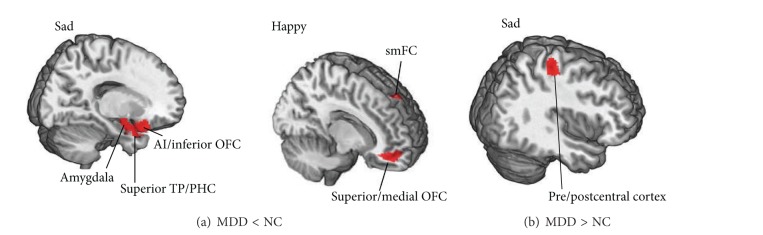
Abnormal regional activity of the MDD patients in early emotion perception at peak time points. Decreased (a) and increased activity (b) in the MDD patients. MDD: major depressive disorder; NC: normal control; AI: anterior insula; OFC: orbitofrontal cortex; TP: temporal pole; PHC: parahippocampus; smFC: superior medial frontal cortex.

**Figure 2:**
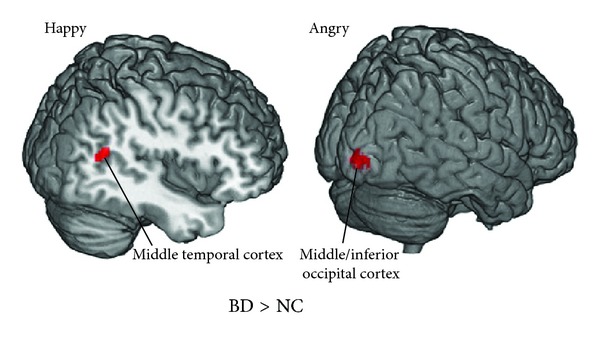
Increased regional activity of the BD patients in early emotion perception at peak time points. BD: bipolar disorder; NC: normal control.

Overall, both patient groups displayed regional gamma hyperactivity compared to the NC group, but only the MDD patients exhibited the diminished gamma activity, as listed in Table 2. Overall, we found diminished gamma responses at very early time points (30–70 ms after stimulus onset) and elevated gamma responses at later time points (80–115 ms). Figures 1 and 2 showed the brain regions with abnormal gamma activity at peak time points (shown in Table 2) in the MDD and BD patients, respectively.

The MDD patient showed decreased gamma responses to sad and happy faces within 70 ms and increased gamma responses to sad faces around 100 ms, relative to the NC group (Table 2). No significant difference of brain responses to angry or neutral faces was found. The decreased gamma responses to sad faces were in the right anterior insula/inferior OFC (40–70 ms), the right superior temporal pole/parahippocampus (55–70 ms), and the right amygdala (55–65 ms). The hypoactivity responding to happy faces was located in the right superior/medial OFC and left superior medial frontal cortex during 30–40 ms. On the other hand, the right precentral/postcentral cortex (95–115 ms) of the MDD patients was more activated to sad faces, compared to the NCs.

As to the BD patients, only increased responses were found in comparison with the NC group, including the right middle temporal cortex (90–100 ms) to happy faces and the right middle/inferior occipital cortex (80–100 ms) to angry faces. (Table 2). No significant difference between the BD and NC groups in response to sad and neutral facial expressions was found.

### 3.1. Correlation between Abnormal Regional GBAI and Symptomatic Data

**Figure 3:**
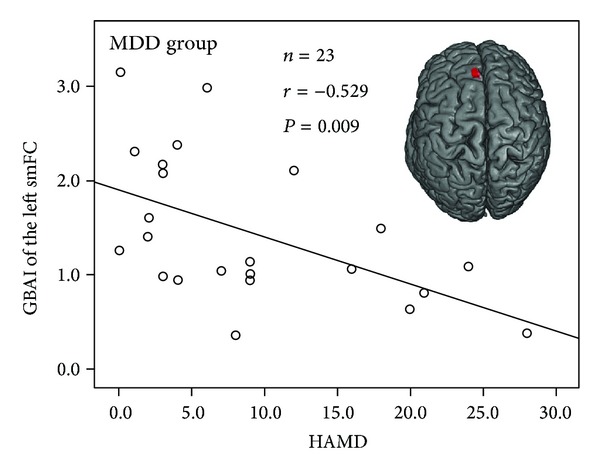
Correlation between HAMD scores and smFC activity of the MDD patients. The HAMD scores of the MDD patients were negatively correlated with the GBAI of the left superior medial frontal cortex (*r* = −0.529, *P* = 0.009 at 35 ms). HAMD: Hamilton Depression Rating Scale; MDD: major depressive disorder; GBAI: gamma-band activation index; smFC: superior medial frontal cortex.

The correlations between the abnormal regional GBAIs and the symptomatic and demographic data of the patient groups were assessed. Only the HAMD score of the MDD patients was found to be negatively correlated with the GBAI of the left superior medial frontal cortex (*r* = −0.441, *P* = 0.035 at 30 ms;* r* < −0.521, *P* < 0.011 at 35 and 40 ms), as shown in Figure 3. In the BD patients, no significant correlation between symptomatic/demographic data and abnormal regional GBAIs was found.

### 3.2. Discriminant Analysis

**Figure 4:**
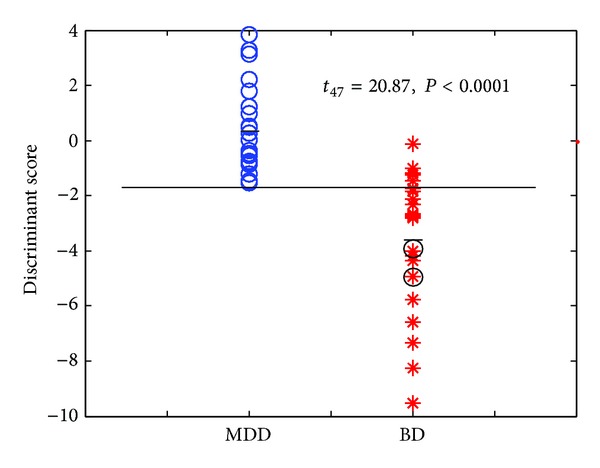
The scatterplots showed individual discriminant function scores of the MDD and BD patients. The black bars illustrated the patient group means. The stepwise discriminant analysis identified four abnormal regional GBAIs of the most discriminating from the MDD (blue circle) and BD (red star) patients (*F*_4,43_ = 9.77, *P* < 0.0001) and the function scores were significantly different (*t*_47_ = 20.87, *P* < 0.0001). The two BD patients (black circle) were initially diagnosed as the MDD patients. The four abnormal regions include the left superior medial frontal cortex (happy 35 ms), the right medial orbitofrontal cortex (happy 35 ms), the right anterior insula/inferior orbitofrontal cortex (sad 60 ms), and the right precentral cortex (sad 105 ms). MDD: major depressive disorder; BD: bipolar disorder; GBAI: gamma-band activation index.

The stepwise linear discriminant analysis identified four brain regions with the most distinguishing capability between the MDD and BD patients (*F*_4,43_ = 9.77, *P* < 0.0001), including the left superior medial frontal cortex (happy 35 ms), the right medial orbitofrontal cortex (happy 35 ms), the right anterior insula/inferior OFC (sad 60 ms), and the right precentral cortex (sad 105 ms). The outputs of the discriminant functions between these two patient groups were significantly different (*t*_47_ = 20.87, *P* < 0.0001).  Figure 4 illustrated the distribution of the discriminant function scores in the MDD (mean = 0.38, se = ±0.32) and BD patients (mean = −3.43, se = ±0.38). The results of leave-one-out cross validation showed that no MDD patient was misclassified and only five out of twenty-five BD patients (80%) were misclassified into the MDD group. Overall, 43 of 48 patients were correctly categorized with a prediction accuracy of 89.6% (Fisher's Exact Test,* P *≤ 3.28 × 10^−9^).

## 4. Discussion 

Our results demonstrated the distinct patterns of gamma oscillatory abnormalities in the MDD and BD patients in responses to emotional facial expression during early perceptual processing. The MDD displayed more deficits in the frontal and limbic regions, including amygdala, OFC, and anterior insula, than the BD. Among these regions, the patterns of gamma activation in the left smFC, right mOFC, right AI/inferior OFC, and the right precentral cortex can accurately classify 89.6% of patients into their diagnosed categories.

Notably, in our study there were two BD patients who were initially diagnosed as the MDD patients and then confirmed as the bipolar disorder two weeks later. Our data from the discriminant analyses showed that these two BD patients were correctly classified using their brain signals measured at the first week (the circle marks in Figure 4) although the patients in this study were not drug-naive or drug-free, which could have a confounding effect on the brain signals. These results suggest that the gamma responses to emotional faces can provide a useful objective index to differentiate the BD patients from the MDD patients and may become a potential biomarker to assist in diagnosis.

Our data showed diminished gamma activity at the amygdala, OFC, and insula in response to sad faces during early emotion perception in MDD patients. This finding may unravel three possible neural mechanisms underlying emotion perception in human brain. First, gamma oscillation could be an emotion-evoked oscillation. Being considered as a mechanism of feature binding [11, 12], early sensory-evoked gamma oscillations were reliably found in various modalities [29]. Early visual evoked gamma-band response within 100 ms after stimulation is proposed to be sensitive to attentional and perceptual factors [30]. The finding of the aberrant gamma responses to emotional faces within 100 ms in the present study indicates that human brain integrates emotional facial features in a very effective and rapid way at the early stage of perceptive processing revealed by gamma oscillations.

Second, the frontoinsular cortex as well as amygdala has been reported to be involved in fast processing of salient information obtained from emotional facial features [31]. The amygdala is proposed to be engaged very early (within 100 ms) in processing negative faces as disclosed by neurophysiological studies using MEG [22] and intracranial electroencephalography [23]. Adolphs [32] reported that humans process emotional facial expressions in perception with simple and highly salient features within 120 ms including the amygdala and OFC. Our finding of diminished gamma activity within 100 ms in the amygdala, anterior insula, and OFC in the MDD patients may indicate their impairments or inefficiency in rapid processing or integrating salient emotion.

Finally, the MDD patients were reported to have a negativity bias (easier to attract patients' attention toward negative stimuli) [9], especially toward sad stimuli. We speculated that the negative-affect bias of depressed patients may be associated with the OFC activity in response to sad facial expressions. The OFC, a key region of the top-down facilitation model, is suggested to rapidly extract low spatial frequency components of inputs (at around 50 ms) from visual or subcortical cortices through the magnocellular route to generate possible candidates of objects [33, 34]. Eliminated OFC activity may result in disrupted top-down information processing. Our results of the diminished activity of the OFC within 50 ms in the MDD patients may suggest a neural evidence of top-down modulation of negative-affect bias in the MDD patients while facing a sad expression.

Our data also indicated that the more severe depression symptoms the MDD patients had, the more eliminated the left smFC responses to happy faces were. Our results were in line with the previous studies [6, 35], which also showed the deactivation of the left prefrontal cortex in depressed patients. Mitterschiffthaler et al. [36] also indicated that reduced medial frontal responses to positive valence stimuli in depressed patients were related to abnormalities of positive emotion processing. Our finding suggests that decreased activity in the smFC underlies irregular positive emotion processing in patients with major depression, which may be one of neural substrate candidates related to anhedonia in depression.

In the BD patients, our finding showed elevated activation in visual regions responded to emotional facial expressions at around 100 ms after visual stimulation. In line with our previous findings of the enhanced occipitotemporal gamma oscillations in the BD patients in sensor-space analysis [8], in this study we found the abnormal occipital or temporal regions of the BD patients when they perceived only the happy and angry faces but not the sad and neutral faces. Happy faces consist of more changeable facial features (e.g., mouth) than negative and neutral faces [37]. Extracting the negative valence from angry expressions is easier than that from sad expressions [38, 39]. Happy and angry faces are high arousal stimuli relative to sad and neutral faces [40, 41]. Hence, the findings of the hyperactivation in the occipital and temporal regions of the BD patients, only to the happy and angry faces but not to the sad and neutral faces, reflect that dysfunction of perceptual processing in the BD patients may be associated with detection of changeable as well as high-arousal facial features. The finding of the hyperactivity of visual regions around 100 ms in the BD patients indicates that the BD patients have altered visual perception ofemotional features which may lead to dysfunction of the subsequent cognitive functions.

Other oscillations (theta, alpha, and beta) play different roles for neuronal functions. A review paper [42] summarizes that theta oscillations are a key mechanism of memory processing in the hippocampus (the main brain area related to theta waves); alpha oscillations are related to function of inhibition in the motor cortex; beta oscillations are associated with functions of motor control and attention in cortical structures. Previous studies indicated that high gamma oscillations (>60 Hz) are related to cognitive and perceptual processes [43, 44], but its significance or role remains unclear [45, 46]. On the other hand, low gamma oscillations (30–50 Hz) have been well documented as a crucial mechanism of perceptual binding and object/face representation in numerous human studies [29, 47–49]. The “binding problem” addresses the physiological mechanisms responsible for combining different features in a visual scene to form a coherent percept [29]. The present study focused on investigating the low gamma responses to emotional faces within 100 ms to reveal the dysfunction of perceptual binding in emotional feature processing of the MDD and BD patients.

## 5. Conclusions 

Our results demonstrated that abnormal activation within 100 ms of the MDD patients in the emotion-related regions (amygdala, inferior/medial OFC, AI, and superior temporal pole) responded to emotional facial expressions, which suggests that the MDD patients may have dysfunctions or negativity biases in perceptual binding of salient emotional features at very early stage. In the BD patients, our finding showed that abnormal activation in visual regions (inferior/middle occipital and middle temporal cortices) responded to emotional facial expressions very early within 100 ms, which supports that the BD patients may hyperactively or sensitively respond to emotional features in perceptual binding. Decreased responses to happy faces in the MDD patients at the left smFC were correlated with their serious depression symptoms, which may support that decreased activity in the smFC underlies irregular positive emotion processing in patients with major depression. The discriminant function of four variables, including gamma activation in the left smFC, right mOFC, right AI/inferior OFC, and the right precentral cortex, accurately classified 89.6% of patients as unipolar/bipolar disorders. These findings indicate different impairments of brain regions in the MDD and BD patients during early facial emotional perception and this abnormal regional gamma activity can be a potential index to distinguish these two mood disorders.

## Figures and Tables

**Table 1:** Demographic variables for bipolar, major depression, and control groups.

	MDD	BD	NC
Gender (male : female)	9 : 14	11 : 14	9 : 15
Age (years)	35.96 (9.58)	36.80 (11.38)	36.62 (11.36)
Age of onset (years)	26.65 (9.26)	28.80 (9.71)	—
Duration of illness (years)	9.30 (7.64)	7.92 (6.18)	—
Number of manic episodes	—	3.08 (2.34)	—
Number of depressive episodes	4.26 (3.61)	4.12 (2.17)	—
Number of major depressive episodes	2.61 (1.08)	3.00 (1.00)	—
Young Mania Rating Scale (YMRS)	0.77 (1.60)	1.44 (2.02)	—
Hamilton Depression Rating Scale (HAMD)	9.09 (8.22)	6.80 (5.29)	—

Except for the gender variable, all other variables are presented as mean (SD).

**Table 2:** Between-group differences (patients versus NCs) in neural response to facial expressions during 30–120 ms.

Group difference	Emotion	Brain region	BA	Time (ms)		Coordinate (mm)	|*t*-value|	Cluster size
				Duration	Peak	*x*, *y*, *z* at peak		
MDD < NC	Sad	R anterior insula/Inferior orbitofrontal cortex	BA47	40–70	60	26, 14, −20	4.94	457
		R superior temporal pole/parahippocampal cortex	BA38	55–70	60	30, 16, −26	4.7	339
		R amygdala		55–65	55	22, −4, −12	4.47	30
	Happy	L superior medial frontal cortex	BA8	30–40	35	−6, 42, 56	4.6	64
		R medial orbitofrontal cortex	BA10/11/32	30–40	35	16, 52, −12	4.46	366
	Angry/neutral	—						
MDD > NC	Sad	R precentral cortex	BA4	95–115	105	22, −24, 62	4.87	217
	Happy/angry/neutral	—						
BD < NC	Sad/happy/angry/neutral	—						
BD > NC	Happy	R middle temporal cortex	BA22	90–100	90	46, −56, 20	4.4	71
	Angry	R middle/inferior occipital cortex	BA18	80–100	95	32, −96, −4	5.15	258
	Sad/neutral	—						

The significant voxels were performed by a one-way ANOVA of three groups (uncorrected *P* < 0.001) and between-group comparisons with a Bonferroni adjustment (uncorrected *P* < 0.000167), all |*t*| values >3.87. The cluster size denotes the number of voxels and coordinates are in MNI space. BA: Brodmann area; R: right; L: left.
